# Comparison of tandem mass spectrometry to fluorimetry for newborn screening of LSDs

**DOI:** 10.1016/j.ymgmr.2017.05.004

**Published:** 2017-06-12

**Authors:** Michael H. Gelb, C. Ronald Scott, Frantisek Turecek, Hsuan-Chieh Liao

**Affiliations:** a Department of Chemistry, University of Washington, Seattle, WA 98195, USA; b Department of Medicine, University of Washington, Seattle, WA 98195, USA; c The Chinese Foundation of Health, Taipei, Taiwan

**Fig. 1:**
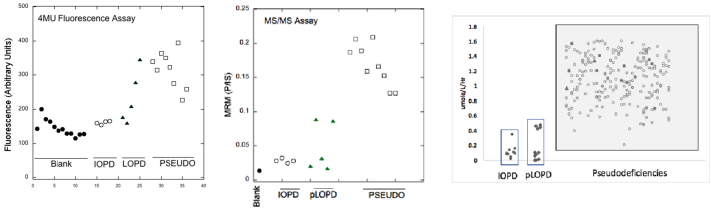
(left) Fluorometer plate reader response for assay of GAA in DBS using the fluorometric assay with 4MU substrate for blank (filled circles), clinically confirmed infantile-onset Pompe patients (IOPD, open circles), potential late-onset Pompe patients (pLOPD, triangles), and pseudodeficiencies (squares). (middle) MS/MS assay of GAA on the same set of DBS (product-to-internal standard ion count ratio). (right) Expanded MS/MS study showing that 96% of 230 pseudodeficiency DBS separate from the IOPD/pLOPD cohort (GAA activity in mole/h/L). From [8].

Sir - while we have no issues with the data presented in the Letter to the Editor [1], we are compelled to give an alternative conclusion. The majority of newborn labs set their own screen cutoffs, and Illinois uses much higher cutoffs than Missouri. It is only meaningful to compare the number of below-cutoff enzyme activity samples with the use of equivalent cutoffs [2], [3], [4]. For example, for mucopolysaccharidosis-I, Missouri steadily lowered their cutoff from 20% to 7% of mean iduronidase activity over the past few years [5], whereas Illinois currently uses a much higher cutoff of 14% [6]. With a 7% cutoff the number of screen positives per 100,000 newborns by digital microfluidics fluorescence in Missouri was 43 [5], whereas tandem mass spectrometry (MS/MS) gave 9 in Washington [4], [6], 12 in Illinois [6], and 16 in New York [6]. For Pompe disease with equivalent cutoffs [4], [6], Missouri reports 52 screen positives per 100,000 [5], Washington 23 [4], [6], Illinois 14 [6], and New York 21 [6]. We hypothesize that the lower number of screen positives with MS/MS is due to the high analytical range (not synonymous with dynamic range) of this method compared to fluorometry [7]. Perhaps the clearest evidence that MS/MS performs better than fluorometry comes from a study in Taiwan using an identical set of DBS showing that MS/MS but not fluorometry separates the pseudodeficiency from the Pompe-affected DBS samples (Fig. 1) [8]. This shows that variation due to DBS sampling is not a dominant effect and disproves the statement that the “analytical/dynamic range is irrelevant” [1]. Additionally, there is no correlation seen in one enzymatic activity compared to another for 111,000 DBS tested for iduronidase, -glucosidase, and -galactosidase [9], showing that variation in white cell count or other DBS quality factors are not dominant effects.
